# A multiplex real-time PCR quantitation of human herpesvirus-6, 7, 8 viruses: application in blood transfusions

**DOI:** 10.1186/s12985-021-01510-6

**Published:** 2021-02-18

**Authors:** Yi Zheng, Youyun Zhao, Yefu Wang, Jun Rao

**Affiliations:** 1 Department of Clinical Laboratory, Hubei Provincial Hospital of Traditional Chinese Medicine, Wuhan, 430061 Hubei China; 2 Hubei Academy of Traditional Medicine, Wuhan, 430074 Hubei China; 3 The State Key Laboratory of Virology, College of Life Sciences, Wuhan University, Wuhan, 430072 Hubei China

**Keywords:** Human herpesvirus, Pityriasis rosea, Blood transfusion, Multiplex real-time PCR

## Abstract

**Background:**

In recent years, fluorescent quantitative polymerase chain reaction assays for detecting viral DNA are in widespread use throughout the world. However, considering the wide distribution of new herpesvirus among the population, we constructed a method to detect HHV-6, 7, and 8 simultaneously.

**Methods:**

The blood samples of 74 blood donors and 45 pityriasis rosea patients were collected. The recombinant plasmids containing U67, U36, and orf65 were constructed to optimize the PCR reaction system. The forward and reverse primers and probe sequences of HHV-6 were as follows: TAAATATCGATGCCGCTCTG, ACGTTCTAGCCATCTTCTTTG, CGCAAACGACAAAGCCA. The forward and reverse primers and probe sequences of HHV-7 were as follows: TTAGACATCTTACACGACAGC, CAGCTTTTCGAACTTGTCAC, TTCATCGGGTACGTCCA. The forward and reverse primers and probe sequences of HHV-8 were as follows: GCGACATATTTCCCTGATCC, CCAACTTTAAGGTGAGAGACC, CATGCGAGCCACCAG. Through the detection of housekeeping genes, DNA sequencing, and optimization of the PCR reaction system, the triple fluorescent quantitative PCR detection system was constructed. Blood samples of blood transfusion staff and pityriasis rosea patients were detected.

**Results:**

The correlations of HHV-6, 7, and 8 between single and multiplex PCR are 0.980, 0.987, 0.965, respectively. In 74 blood donor samples, 16.2% of HHV-6 and 55% of HHV-7 were positive (viral load > 3 log10 copies/ml) according to multiplex real-time PCR. In 45 patients suspected of pityriasis rosea (PR) infection, 40% HHV-6, 73.3% positive cases are found.

**Conclusion:**

With the safety of blood transfusion being a major concern of the public, this method will show good specificity and sensitivity in blood transfusion screening.

## Introduction

According to the WHO's recommendations, each country should formulate a national policy on blood screening all whole blood and apheresis donations for transfusion-transmissible infections [1]. Blood transfusions save lives, but risks are accompanied. All donated blood is subjected to quality-assured screening for transfusion-transmissible infections, including HIV, hepatitis B, hepatitis C, Treponema pallidum (syphilis) and, where relevant, other infections that pose a risk to the safety of the blood supply, such as Trypanosoma cruzi (Chagas disease) and Plasmodium species (malaria)], as well as tests for blood groups and compatibility [2].

Transfusion-associated fatal diseases were once thought to be a clinical phenomenon in profoundly immunosuppressed individuals. The hospital transfusion laboratory needs to take the epidemiology of infection among the population into consideration. Although some routine diagnoses are set for blood donors, "inevitable" factors still exist. Based on the current understanding of the human herpesvirus (HHV), it is necessary to construct a herpesvirus screening system.

HHV is a series of prevalent viruses with widespread infection among the population. Among them, Roseoloviruses can exist in a latent form after primary infection and can be reactivated during immunosuppression periods, particularly in immunodeficient recipients and patients suffering from nervous system diseases [3–5].

Many novel herpesviruses have been described [6–8]. These viruses are Roseola viruses, including HHV-6, 7, and 8. HHV-6 and HHV-7 are ubiquitous, lymphotropic herpesviruses, and are classified along with cytomegalovirus (CMV) into the Betaherpesvirinae subfamily. HHV-8 is categorized into Gammaherpesviruses along with Epstein–Barr virus (EBV).

Multiple lines of clinical and experimental evidence suggest that HHV-6 and HHV-7 may act as an accelerating factor, especially in the immunocompromised host [9–11]. As early as in 2006, Engels and his colleagues provided evidence of HHV-8 transmission by blood transfusion [12], suggesting that HHV-8 blood screening for immunocompromised patients should be warranted. Higher morbidity and mortality rates in transfused patients are often associated with reactivated herpesvirus infections [13].

To construct and perfect a screening system for HHV-6, 7, and 8, we studied 74 samples from healthy blood donors and 45 samples from patients suspected of pityriasis rosea (PR) infection using the multiplex real-time PCR assay.

## Materials and study design

### Strains

For assay calibration, HHV-6 (U1102 type A strain and Z29 type B strain), HHV-7 (RK strain), HHV-8 (GK strain) standard strains were obtained from the American Type Culture Collection (ATCC; Maryland, USA). These standard strains were then used to obtain recombinant plasmid and test the specificity of the primers and probes.

Additionally, human CMV and Herpes simplex virus 1/2 (HSV-1/2) standard strains obtained from the Hubei Provincial Center For Disease Control and Prevention (HBCDC, Wuhan, Hubei, China) were used as negative controls.

Due to a few research institutes focusing on HHV-6, 7, and 8 in China, we have amplified specific target gene segments of HHV-6, -7, and -8 from standard strains. We treated HHV-6, 7, and 8 plasmids at 10^6^ concentrations as positive controls. The transfection and transformation processes have been mentioned briefly below. The three plasmids were used to test the reproducibility of the multiplex real-time PCR assay.

### Clinical specimens sample collection

Blood samples from 74 blood donors were collected between August 2019 and December 2019 from the blood donation center in Hubei Provincial Hospital of Traditional Chinese Medicine (Wuhan, Hubei, China). Also, another 45 blood samples from adults suspected of having PR infection were enrolled in this study. The patients were selected based on the inclusion criteria (skin lesion on the trunk or limbs that could be a 2 ± 1 cm oval scaly plaque with a central salmon-colored area or numerous smaller [14], oval, erythematous, scaly, and slightly pruritic plaques).

In total, this prospective study was performed on 119 blood samples (74 from blood donors and 45 from patients suspected of PR infection). All participants signed the informed consent for the study. All the blood samples were stored in tubes containing sodium citrate as an anticoagulant.

To avoid the impact of hemolysis on DNA extraction, we didn't store the whole blood at a low temperature. As an alternative, DNA was extracted immediately and then stored at − 70 °C.

### DNA extraction

Particular protection measures were taken to avoid sample-to-sample contamination: different rooms and dedicated equipment were used for DNA extraction and processing. Furthermore, for PCR setup and analysis, all pipette tips had filters for aerosol protection.

One milliliter of each whole blood sample was used to extract the DNA template. According to the manufacturer's protocols, DNA extraction was performed using the QIAamp DNA Blood Midi Kit (QIAGEN, Shanghai, China). All the hematocrit of blood samples were within the normal range (male 0.40–0.50, female 0.37–0.45). The viral DNA of the standard strains of HHV-6, 7, and 8 were extracted using the QIAamp 96 Virus Kit (QIAGEN, Shanghai, China).

After extraction, all the DNA was suspended with 50 μl of sterile water. Finally, the DNA was stored at -70 ℃ until PCR detection.

### Primers and probes

According to previous studies [15, 16], U67, U36, and ORF65 were chosen as the target gene for HHV-6, 7, and 8, respectively. U67 sequences of HHV-6A and HHV-6B, U36 sequences of HHV-7, and ORF65 sequences of HHV-8 were downloaded from the NCBI GenBank sequence database (http://www.ncbi.nlm.nih.gov).

Primers and TaqMan-LNA probes for HHV-6, 7, and 8 were designed using the beacon designer (PREMIER Biosoft International, Palo Alto, CA). Although HHV-6, 7, and 8 were exogenous genes, the human CCR5 gene was still chosen for amplification as a "house-keeping" gene and used as an internal control to assure the quality of the extracted DNA in each sample and its suitability for PCR. According to Broccolo's report [17], forward primer 5′-CAAAGCCAATTATCCAGAGCG-3′, reverse primer 5′-CGCTGGTTGAGGATGATCGA-3′, probe 5′-6FAM-TACGCAACGC CAACAGACCTAGCGA-BHQ-3'.

The primers and TaqMan-LNA probes for HHV-6, 7, and 8 (Table 1) were synthesized by Shanghai Sangon Biotechnology Co., Ltd. (Shanghai, China). Their specificity was checked by BLAST search through the database (http://blast.ncbi.nlm.nih.gov/). No significant similarities were found.

**Table 1:** The oligonucleotide sequences of primers and TaqMan-LNA probes sequences

Virus type	Target gene (NO^a^)	Sequence (5′ → 3′) (forward and reverse primer, probe)	Positions^b^ (position of HHV-6B)	Size^c^ (bp)	Fluorophore label
HHV-6	U67(NC_001664^d^)	TAAATATCGATGCCGCTCTG	102,708–102,727 (104,006–104,025)	76	6FAM-BHQ-2
	(NC_000898^e^)	TACGTTCTAGCCATCTTCTTTG	102,762–102,783 (104,060–104,081)		
		CGCAAACGACAAAGCCA	102,735–102,751 (104,033–104,049)		
HHV-7	U36(NC_001716)	TTAGACATCTTACACGACAGC	55,407–55,427	147	JOE-BHQ-2
		CAGCTTTTCGAACTTGTCAC	55,534–55,553		
		TTCATCGGGTACGTCCA	55,513–55,529		
HHV-8	ORF65(AF148805)	GCGACATATTTCCCTGATCC	112,438–112,457	108	Cy5-BHQ-2
		CCAACTTTAAGGTGAGAGACC	112,525–112,545		
		CATGCGAGCCACCAGG	112,466–112,481		

*bp* base pair

^a^NCBI Genbank accession number

^b^Complete genome nucleotide positions

^c^Size of PCR products

^d^NCBI Genbank accession number of HHV-6A

^e^NCBI Genbank accession number of HHV-6B

### Recombinant plasmids

The plasmids of HHV-6, 7, and 8 (containing 76 bp U67 amplicon, 147 bp U36 amplicon, and 108 bp ORF65 amplicon, respectively) and CCR5 (containing 133 bp CCR5 amplicon) were obtained from Shanghai Sangon.

The plasmids mentioned above were used to construct standard curves for HHV-6, 7, and 8 in the single real-time PCR and multiplex real-time PCR. The human CCR5 gene's plasmids were amplified in every assay, indicating that no total inhibition occurred during the nucleic acid amplification reaction and normalization of the HHV-6, 7, and 8 viral loads.

### Single real-time PCR

To expand and quantify the plasmids, we prepared competent *E.coli* DH5α, in which the plasmids were propagated. Four plasmids (HHV-6, 7, 8, and CCR5) were purified using the QIAamp plasmid Midi Kit and quantified by measuring optical density (OD)260 using an ultraviolet spectrophotometer (SHIMADZU, Tokyo, Japan). The copies of the extracted plasmids were calculated using the formula: 6.02 × 10^23^ (copies/mol) × A260 (ng/ml) ÷ (DNA length × 660) = copies/ml. A tenfold series dilution of plasmids from 1 × 10^7^ to 10^2^ copies/μl was amplified to assess the detection limit and establish a standard curve. Each dilution (5 μl) was also added to the single real-time PCR reaction mixture.

To exclude the possibility of contamination during the PCR, positive controls (10^6^ copies/well plasmids of HHV-6, 7, 8), negative controls (DNA extracted from HSV-1, 2, CMV and sterile water) and standard DNA from 1 × 10^7^ to 10^2^ copies/reaction were amplified in each experiment.

To optimize the PCR condition, concentrations of each reagent should be normalized (Table 2). After optimization of the reaction conditions, PCR of each virus was carried out under corresponding conditions. The extracted DNA (5 μl) was added to the reaction mix to achieve a final reaction volume of 50 μl. Each sample was analyzed in duplicate for each virus.

**Table 2:** Optimization of PCR conditions

Type of ingredient	Gradient (variation rate)		Optimal ultimate concentration (type of virus)	
Mgcl_2_^a^	2.5 mM–5.5 mM (0.5)	3.5 mM (HHV-6)	4 mM (HHV-7)	3.5 mM (HHV-8)
Deoxynucleotide mixture^a^	0.14 mM–0.32 mM (0.02)	0.16 mM (HHV-6)	0.16 mM (HHV-7)	0.16 mM (HHV-8)
Primer^a^	0.26 μM–0.35 μM (0.01)	0.29 μM (HHV-6)	0.29 μM (HHV-7)	0.31 μM (HHV-8)
Probe^a^	0.12 μM–0.40 (0.02)	0.24 μM (HHV-6)	0.26 μM (HHV-7)	0.24 μM (HHV-8)
Taq DNA polymerase^a,b^	0.1U–0.6U (0.1)	0.5U (HHV-6)	0.6U (HHV-7)	0.6U (HHV-8)

^a^Initial concentration of Mgcl_2_, deoxynucleotide mixturea, primer, probe, Taq DNA Polymerase is 25 mM, 10 mM, 10 μM, 10 μM, 5U/μL respectively

^b^Taq DNA Polymerase was obtained from TaKaRa Biotechnology Dalian Co. Ltd., Liaoning, China

The annealing temperature was optimized at 55, 58, 61, and 64 °C while time was optimized for 25, 30, 35, 40, 45, 50 s. The optimal cycling condition was 94 °C for 10 min, followed by 40 cycles of amplification at 94 °C for 30 s and then 58 °C for 40 s. Results were expressed as the number of genome equivalent copies (EqCop) per ml of whole blood.

### Multiplex real-time PCR

The standard DNA from 1 × 10^7^ to 10^2^ copies were mixed per reaction and used to construct the standard curves. Similarly, the negative controls (DNA extracted from HSV-1, 2, HCMV, and sterile water) were amplified for every five samples in each experiment, consisting of all reagents except for the standard DNA sample.

According to the results of the single PCR optimization, the multiplex real-time PCR was performed in reaction volumes of 50 μl including PCR (1 ×) buffer, 4 mM MgCl_2_, 0.48 mM deoxynucleotide mixture, 0.5 U Hotstart Taq DNA Polymerase, 0.29 μM HHV-6 primer, 0.29 μM HHV-7 primer, 0.31 μM HHV-8 primer, 0.24 μM HHV-6 probe, 0.26 μM HHV-7 probe, 0.24 μM HHV-8 probe and 5 μl DNA template.

For the final calculation of the DNA copy number, we generated a standard curve of known amounts (10^2^ to 10^7^ copies) of plasmids isolated and quantified from HHV-6 (variant A U1102 strain and variant B Z29 strain), 7 (RK strain), 8 (GK strain). Each point was triplicate, and the standard curve for each one was repeated. The final calculation of the DNA copy number for each sample and virus was performed by the software provided by the ABI Prism 7500 instrument.

### DNA sequencing

According to S.Yalcin's description [18], HHV-6 DNA was amplified by nested PCR, basically as described previously. The PCR was designed to amplify the IE (U90) regions of HHV-6. The primers for the first PCR were 5′-TTCTCCAGATGTGCCAGGGAAATCC-3′ and 5′-CATCATTG TTATCGCTTTCACTCTC-3′. The primers for the second PCR were 5′- AGTGACAGATCTGGGC GGCCCTGATAACTT-3′ and 5′-AGGTGCTGAGTGATCAGTTTCATAACCAAA-3′. The PCR products' size was 506 bp (the first run) and 363 bp (the second run). Each sample (5 μl) was added to 45 μl of reaction mixture containing 32.5 μl sterile water, 5 μl KOD buffer, 0.2 mM deoxynucleotide mixture, 200 nM primers, and 1.25 U KOD DASH DNA Polymerase (Promega, Madison, WI). The first PCR cycling conditions comprised of 94 °C for 6 min, followed by 30 cycles of amplification at 94 °C for 30 s, 62 °C for 5 s, and 72 °C for 15 s. The conditions were the same as the first PCR, except that the inner primers were used instead.

According to Yalcin's description [18], HHV-7 DNA was amplified by nested PCR as described previously. The PCR was designed to amplify the major capsid protein gene of HHV-7. The primers for the first PCR were 5′-AGTTCCAGCACTGCAATCG-3′ and 5′-CACAAAAGCGT CGCTATCAA-3′. The primers for the second PCR were 5′-CGCATACACCAACCCTACTG-3′ and 5′-GACTCATTATGGGGATCGAC-3′. Each sample (5 μl) was added to 45 μl of reaction mixture containing 32.5 μl sterile water, 5 μl KOD buffer, 0.2 mM deoxynucleotide mixture, 200 nM primer, and 1.25 U KOD DASH. The first PCR cycling conditions comprised of 94 ℃ for 6 min, followed by 30 cycles of amplification at 94 °C for 30 s, 60 °C for 5 s, and 72 °C for 16 s. The second round of 40 cycles was performed with inner primers.

According to Tisdale et al.'s description [19], HHV-8 DNA was amplified by nested PCR, basically as described previously. The PCR was designed to amplify the ORF 75 region of HHV-8. The primers for the first PCR were 5′- TATTCGCGGCCTTGGCAACC-3′ and 5′-AAGATGCGCA CCGCGTTGTC-3′. The primers for the second PCR were 5′-ACGTACAGCAGGCCGAGATG-3′ and 5′- GGAGCTGTCGCGATAGAGGT-3′. Each sample (5 μl) was added to 45 μl of reaction mixture containing 32.5 μl sterile water, 5 μl KOD buffer, 0.2 mM deoxynucleotide mixture, 200 nM primer, and 1.25 U KOD DASH. The first PCR cycling conditions comprised of 94 °C for 6 min, followed by 40 cycles of amplification at 94 °C for 30 s, 66 °C for 8 s, 72 °C for 15 s. The second PCR cycling conditions comprised of 94 °C for 6 min, followed by 40 cycles of amplification at 94 °C for 30 s, 59 °C for 5 s, and 72 °C for 12 s.

The PCR products were resolved and detected visually using 0 ± 8% agarose gel electrophoresis with ethidium bromide staining. The amplification products were 423, 264, and 245 bp in length for HHV-6, 7, and 8. The PCR products of HHV-6, 7, 8 DNA, together with corresponding inner forward primers, were provided by Sangon. DNA sequencing was performed in single directions. Sequences were aligned, checked, and corrected for the analyzer's possible mistakes using the program Mega 3.1 software (Mega Software, Tempe, AZ, USA). Comparison with the reference strains in the GeneBank database was made at the NCBI Blast programs.

### Statistical analysis

Based on the HHV-6,-7,-8 plasmid standard curve spanning from 10^7^ to 10^2^ copies, the viral load was calculated and expressed as copies/ml. Correlation coefficient (R) values of standard curves were calculated using software supplied by the ABI PRISM 7500. Clinical sensitivity, specificity, absolute agreement and kappa values of the assay, the coefficient of variation of the standards, and the correlation between the multiplex and the single real-time PCR assays for viral load were calculated using the SPSS 16.0 statistical package (SPSS Inc., Chicago, USA). All tests were conducted at the 5% level of significance. Due to the limited sample size, only descriptive statistics were provided.

## Results

### Internal control detection

The reproducibility of the method was detected by internal control. While the specimens were carried out, identical experiments were also done using internal control primers for CCR5. The CCR5 gene was detected in all the examined samples, with CT values below the threshold value of 35. Therefore, significant DNA losses did not occur during nucleic acid extraction. The determination by real-time PCR with CCR5 as an internal control was highly reproducible. When comparing the amount of CCR5 in healthy blood donors with that in suspected pityriasis rosea patients, no difference was observed *P* > 0.05 (data not shown).

### Specificity of real-time TaqMan-LNA PCR assays and sequence identification

As described previously, positive controls (10^6^ copies plasmid of HHV-6, 7, 8) and negative controls (DNA extracted from HSV-1, 2, CMV, and sterile water) were amplified in each experiment. The specificity of the PCR products was confirmed by gel-based post-PCR analysis. Real-time TaqMan-LNA PCR assays applied to either the HSV-1, 2, or CMV led to negative results (i.e., CT values > 35), indicating the specificity for HHV-6, 7, 8 (data not shown).

After real-time PCR, the products of standard plasmids of HHV-6,-7,-8 were also confirmed by electrophoresis. However, due to electrophoresis's limitations, only the high concentration plasmids (> 10^4^ copies) were visible. According to DNA sequencing, the gene fragments of HHV-6 (U67), HHV-7 (U36), and HHV-8 (ORF65) had a 100% similarity with the corresponding GenBank sequence of each gene. The products of the positive specimens also had a 100% similarity.

### Standard curves and dynamic range of multiplex real-time PCR assays

**Fig. 1:**
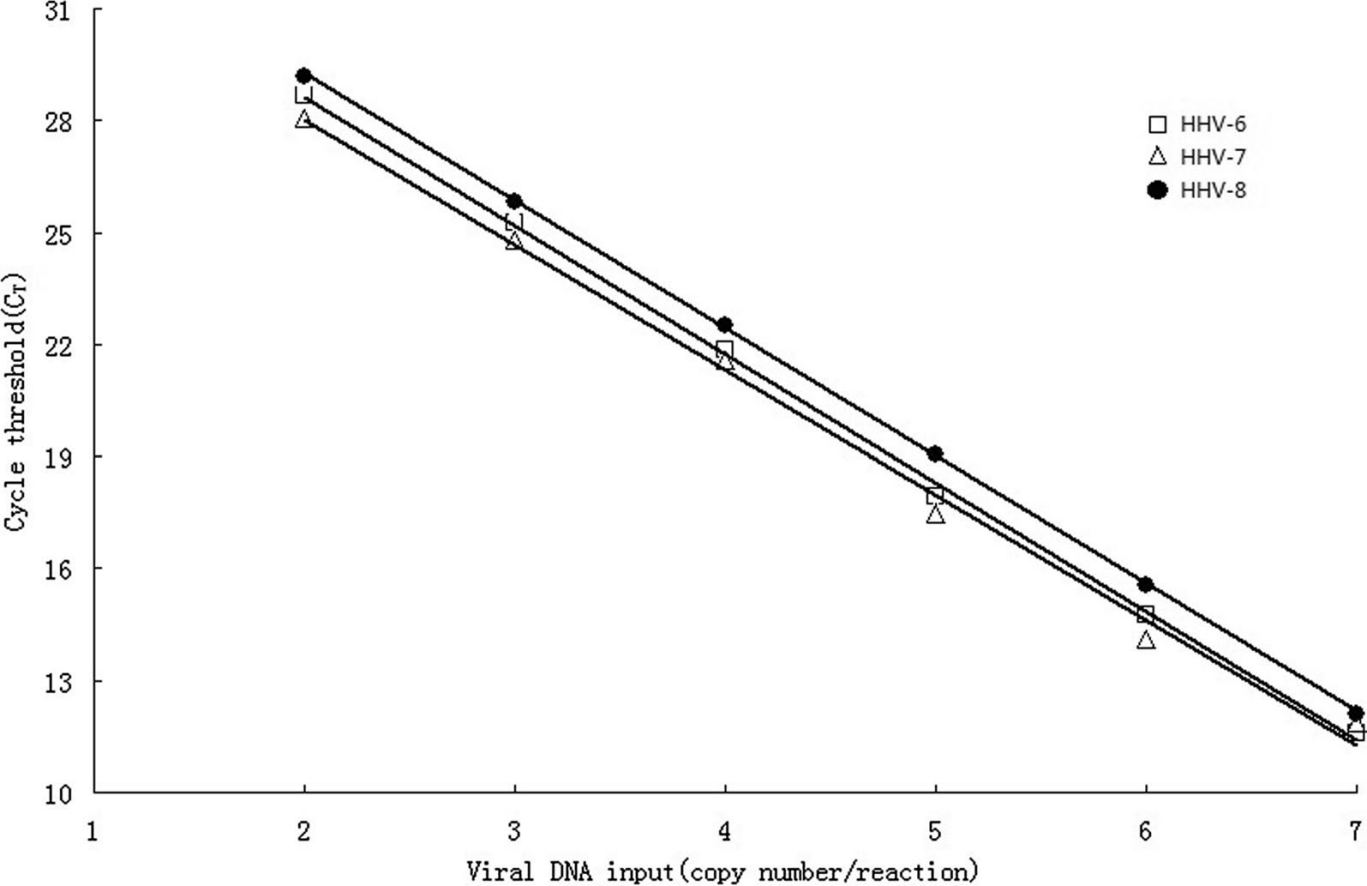
Standard curves for HHV-6, 7, 8 using real-time PCR.C_T_ values were plotted against viral DNA input (copy number per reaction). The copy was expressed as Log_10_

To construct standard curves, serial tenfold dilutions of HHV-6,-7,-8 standard DNA, ranging from 10^7^ to 10^2^ copies per well, were tested to determine the linearity and the sensitivity of multiplex real-time PCR assays. For each point, CT values were measured in triplicate. The dynamic range was approximately 6 logs (1.0^2^ to 10^7^ copies) with a correlation coefficient of 0.9991 (HHV-6), 0.9951 (HHV-7), and 0.9926 (HHV-8). As expected, every ten-fold decrease in the copy number affected an average increase of -3.4 in the CT value (-3.45 for HHV-6, -3.34 for HHV-7, and -3.41 for HHV-8) (Fig. 1). Real-time PCR reactions for HHV-6, HHV-7, and HHV-8 had similar sensitivities, and the limit of detection allowed 100 copies of the target gene per well.

### Dynamic range of nested PCR

To compare the sensitivity of multiplex real-time PCR with nested PCR, the positive controls (10^6^ copies plasmids of HHV-6, 7, 8) were diluted into 1, 2, 3, 4, 5, and 6 (log10 copies/reaction) folds. The identical copies of DNA were amplified in both nested PCR and real-time PCR assays. The target gene beyond 10^3^ copies was shown after the first round PCR, and 10^1^ copies gene were shown after the second round PCR.

### Influence of single and multiplex real-time PCR assays

To investigate the influence between single and multiplex real-time PCR assays for a single virus, serial tenfold dilutions of HHV-6, 7, 8 standard DNA, ranging from 10^7^ copies to 10^2^, were used as both single and multiples real-time PCR templates. Each point was triplicate to avoid data fluctuation. The average data were shown in Table 3. The correlation between the multiplex real-time PCR and single real-time PCR in viral load was not shown in this table. The correlation of HHV-6, -7, -8 between single and multiplex PCR was 0.980, 0.987, and 0.965, respectively, suggesting that there was almost no variation between them.

**Table 3:** Comparison single PCR with multiplex PCR

Subject	2^c^	3	4	5	6	7	Paired T values^a^	Sig. (2-tailed)^b^
HHV-6 (single PCR)	28.42^d^	24.87	21.74	17.63	14.45	11.31		
(multiplex PCR)	28.52	24.90	21.76	17.87	14.56	11.35	− 2.673	0.44 > 0.05
HHV-7 (single PCR)	28.05	24.81	21.56	17.45	14.11	11.87		
(multiplex PCR)	28.14	24.83	21.75	17.56	14.14	11.93	− 3.266	0.22 > 0.05
HHV-8 (single PCR)	29.20	25.82	22.52	19.05	15.57	12.12		
	29.35	25.93	22.68	19.26	16.03	12.21	− 3.553	0.16 > 0.05

^a^Paired T test was shown

^b^Sig(2-tailed) is the P value’s name in SPSS

^c^The viral loads are expressed as log^10^ copies/reaction

^d^The data of CT value were shown

### Application in blood donors and clinical samples

**Fig. 2:**
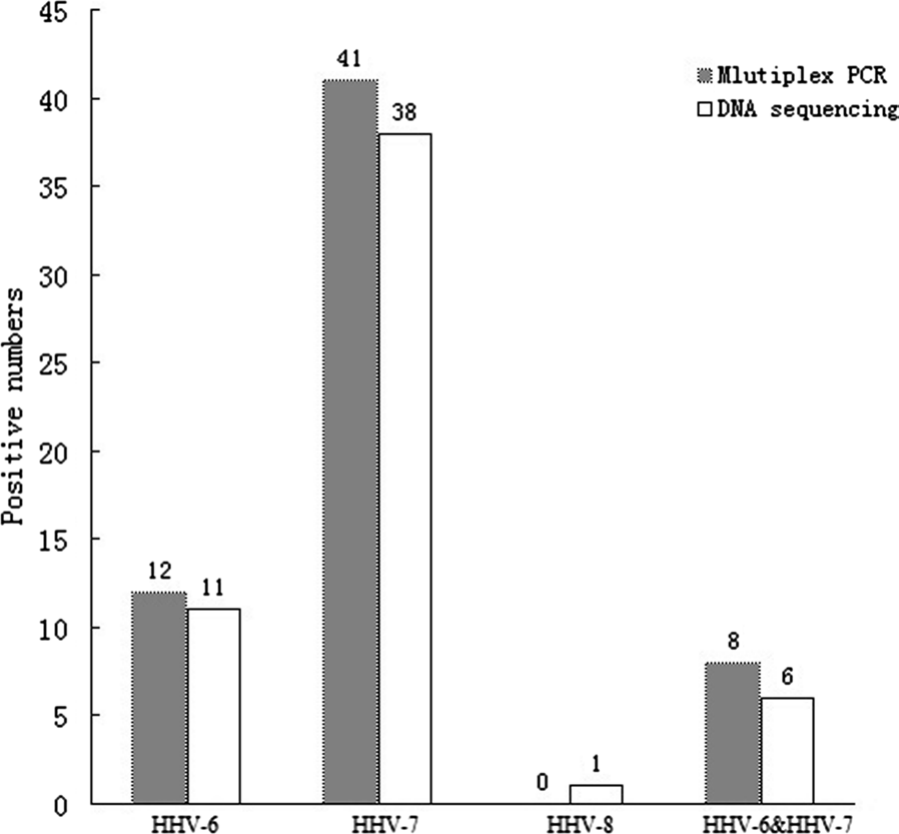
Comparison of multiplex real-time PCR with DNA sequencing (blood donors)

**Fig. 3:**
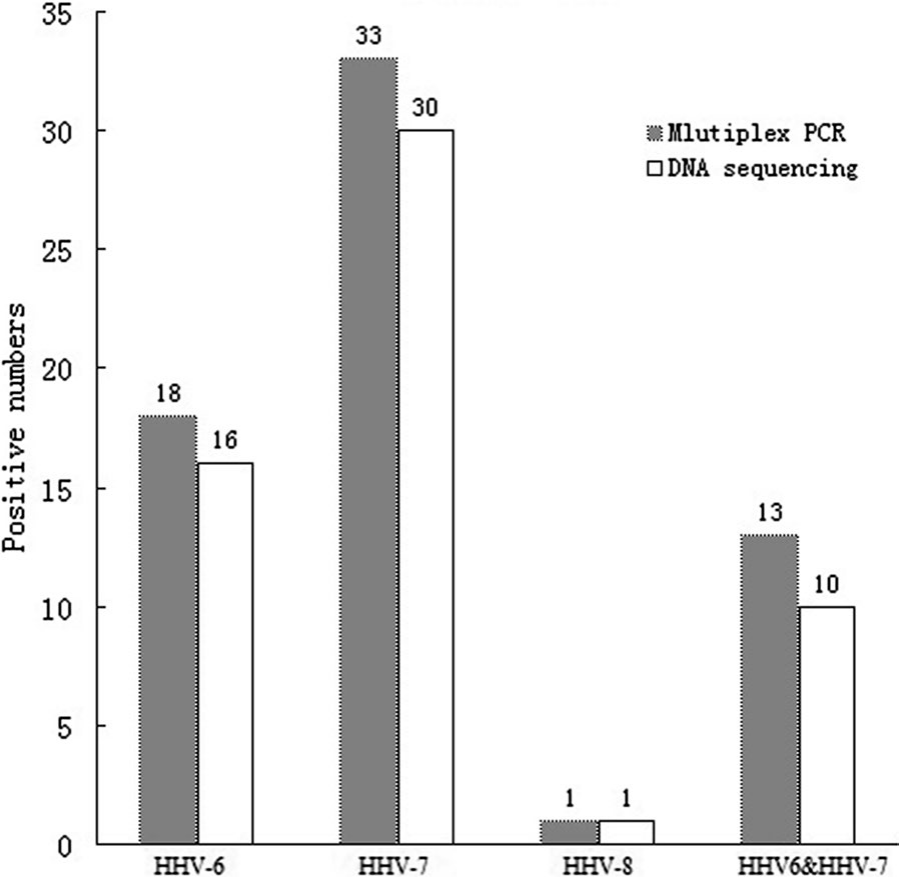
Comparison of multiplex real-time PCR with DNA sequencing (clinical samples)

A total of 119 samples was processed. Each whole blood sample was in duplicate, one for DNA sequencing and one for multiplex PCR. The comparison of multiplex real-time PCR with DNA sequencing is shown in Fig. 2 (blood donors) and Fig. 3 (Clinical samples). To compare the multiplex real-time PCR and DNA sequencing in samples, we used sensitivity, specificity, and Youden index as reference indexes, and data are shown in Table 4.

**Table 4:** Comparison multiplex real-time PCR with DNA sequencing

Subject	No. (%) positive numbers			
	HHV-6	HHV-7	HHV-8	HHV-6&HHV-7
Multiplex PCR (blood donors)	12 (16.2)	41 (55.4)	0 (0)	8 (10.8)
DNA sequencing (blood donors)	11 (14.8)	38 (51.3)	1 (1.3)	6 (8.1)
Sensitivity (blooddonors)	100%	100%	0%	100%
Specificity (blood donors)	98.4%	91.8%	100%	97.1%
Multiplex PCR (clinical samples)	18 (40)	33 (73.3)	1 (2.2)	13 (28.8)
DNA sequencing (clinical samples)	16 (35.5)	30 (66.7)	1 (2.2)	10 (22.2)
Sensitivity (clinical samples)	100%	100%	100%	100%
Specificity (clinical samples)	93.1%	80%	100%	91.4%

In 75 blood donors samples, 16.2% of HHV-6 and 55% of HHV-7 were positive (viral load > 3 log10 copies/ml) by multiplex real-time PCR. A total of 8 co-infection (HHV6 and HHV-7) cases were also detected. It is worth mentioning that the 2 Western Blot samples had high HHV-6 viral loads (> 7 log10 copies/ml) with a median of 7.3 (range from 7.2 to 7.4 log10 copies/ml) in 75 WB samples. Using the DNA sequencing method, we confirmed that all the positive donors were the HHV-6B variant.

In 45 patients suspected of PR infection, 40% HHV-6, and 73.3% positive cases were found. Furthermore, one HHV-8 positive sample was detected. The viral load of positive cases in clinical samples was 5.6 log10 copies/ml, while the viral load of blood donors was 4.2 log 10 copies/ml (*P* < 0.05). Therefore, we concluded that there were statistically significant differences between viral loads in blood donors and PR patients.

## Discussion

In general, HHV-6 DNA in plasma or serum is considered a good marker for active infection. Recent studies have shown that HHV-6 DNA in plasma does not necessarily reflect the amount of virus produced by the active infection of distant lymphoid tissue and organs [20]. Hence, for the conservative purposes of this study, only results from whole blood samples were analyzed.

We found 2 high viral load cases of HHV-6 (> 7 log10 copies/number), which was considered as Chromosomally Integrated Human Herpesvirus 6 Genome [21].

In recent years, real-time PCR has been widely applied in the diagnosis of β- herpesviruses. TaqMan-LNA probe is a new type of nucleic acid analog containing a 2′-oxo-4′ carbon methylene connection. It limits the flexibility of the ribofuranose ring in this connection and becomes a rigid bicyclic. Through these means, real-time PCR has improved hybridization efficiency and stability. Compared with normal DNA fluorescent probes, a single LNA molecule can lead to a melting point up to 8℃. For the above reasons, a real-time PCR assay was designed based on a Taq-Man LNA probe and applied in screening HHV-6, 7, 8 in healthy donors.

In our research, the standard curves for HHV-6,-7,-8 were created and showed excellent linearity and correlation. As shown by the comparison results of multiplex real-time PCR with single real-time PCR, no obvious difference was found (*P* > 0.05).

Due to privacy considerations, it is difficult to collect cases in this study, and the number of cases is small. Especially for HHV-8 patients, it is difficult to collect. Some reports show that HHV-8 patients generally exist in China's ethnic minority areas. Therefore, the author analyzed HHV-8 positive in this study caused by the contamination of positive plasmid.

## Conclusion

Nowadays, the diagnostic means applied clinically aimed at HHV are almost blank. The means of simultaneous diagnosis of HHV-6, 7, and 8 are even fewer. Our multiplex real-time PCR will have a vast potential for future development. In our report, only preliminary results were shown. Next, we plan to do some substantive research by detecting more clinical samples for monitoring HHV-6, 7, and 8.

## Data Availability

Not applicable.

## Abbreviations

CMV: Cytomegalovirus
EBV: Epstein-Barr virus
FQ-PCR: Fluorescent quantitative polymerase chain reaction
HHV: Human herpesvirus
PR: Pityriasis rosea
